# *FASN*-Mediated Lipid Metabolism Regulates Goose Granulosa Cells Apoptosis and Steroidogenesis

**DOI:** 10.3389/fphys.2020.00600

**Published:** 2020-06-26

**Authors:** Xi Chen, Kailiang Huang, Shenqiang Hu, Gang Lan, Xiang Gan, Shanyan Gao, Yan Deng, Jiwei Hu, Liang Li, Bo Hu, Hua He, Hehe Liu, Lu Xia, Jiwen Wang

**Affiliations:** ^1^Farm Animal Genetic Resources Exploration and Innovation Key Laboratory of Sichuan Province, College of Animal Science and Technology, Sichuan Agricultural University, Chengdu, China; ^2^Key Laboratory of Agricultural Information Engineering of Sichuan Province, College of Information Engineering, Sichuan Agricultural University, Ya’an, China

**Keywords:** lipid metabolism, granulosa cells, *FASN*, apoptosis, progesterone

## Abstract

Lipid metabolism participates in regulating the functions of granulosa cells (GCs), which is important for follicular development. In this experiment, goose GCs from pre-hierarchical follicles and hierarchical follicles were selected to be the model for studying the putative regulatory role of lipid metabolism in apoptosis and steroidogenesis, through overexpression and interference with fatty acid synthase (*FASN*). When *FASN* was overexpressed, the lipid accumulation was increased in hierarchical GCs (hGCs) and it was increased in the two categorized GCs when *FASN* was interfered. In addition, the apoptosis of the two categorized GCs was increased when *FASN* was overexpressed, and their progesterone production was decreased when *FASN* was interfered. The results of qRT-PCR showed that, when *FASN* was overexpressed, the expression level of *CYP11A1* was decreased in pre-hierarchical GCs (phGCs), while the expression levels of *SCD1*, *DGAT2*, *APOB*, and *StAR* were increased in hGCs. When *FASN* was interfered, the expression levels of *CPT-1*, *DGAT2*, and *StAR* were decreased whereas the expression level of *CYP11A1* was increased in phGCs, and the expression levels of *CPT-1*, *SCD1*, and *StAR* were decreased in hGCs. These results not only identify the different effects of manipulated *FASN* expression on lipid metabolism of goose phGCs and hGCs but also demonstrate that *FASN*-mediated lipid metabolism plays an important role in regulating apoptosis and steroidogenesis of *in vitro* cultured goose GCs.

## Introduction

Female fertility depends on the normal development of ovarian follicles. As the key follicular component, granulosa cells (GCs) are essential for follicular growth, maturation, and ovulation due to their pivotal roles in regulating cellular apoptosis and steroidogenesis. In recent years, increased attention has been paid to lipid metabolism in GCs, and there are evidences that lipid metabolism in GCs is essential for maintaining ovarian follicle development in humans (Hu and Qiao, 2011) and other mammals (Campbell et al., 2010; Elis et al., 2015). Nevertheless, very little is known about the underlying mechanisms by which lipid metabolism in GCs modulates vertebrate ovarian functions. In contrast to the situation in mammals, lipid metabolism in GCs of avian may be more crucial and complicated because of the deposition of large amounts of liver-synthesized yolk precursors (mainly lipids) as the follicles develop. Besides, several lines of evidence showed that *de novo* lipogenesis (DNL) exists in the avian ovary including GCs, which may play important roles in regulating ovarian activities (Alvarenga et al., 2011). In support of this, our previously published study has demonstrated that fatty acid synthase (*FASN*), a key player in DNL, exhibited a stage-dependent expression pattern in GCs from goose different-sized follicles (Wen et al., 2019). Thus, we hypothesized that *FASN*-mediated lipid metabolism could be involved in regulating follicle development. More importantly, lipid metabolism dysfunction in GCs has been reported to be related to several ovarian diseases, such as GC tumor (Leung et al., 2019). Therefore, it is of great value to fully clarify the role of *FASN*-mediated lipid metabolism in GCs.

Lipids are not only the energy source and cell membrane components but also signal molecules that participated in the regulation of gene expression. Appropriate amount and composition of lipids are beneficial to cells; otherwise, cell dysfunction can be induced. Lipid metabolism has been associated with intracellular cholesterol concentration, since the inhibition of *FASN* by C75 (an inhibitor of *FASN*) significantly reduced cholesterol biosynthesis in cultured human macrophages (Rae and Graham, 2008). *In vivo*, cholesterol for steroidogenesis comes from the hydrolysis of cholesteryl ester in plasma lipoproteins, whereas *in vitro*, GCs may contribute to the supply of cholesterol since inhibition of cholesterol biosynthesis by simvastatin inhibited progesterone (P_4_) biosynthesis in cultured bovine GCs (Spicer et al., 1996). A previous study in bovine GCs has suggested that inhibiting *FASN* by C75 reduced P_4_ biosynthesis (Elis et al., 2015). In addition, several studies have also correlated the process of lipid metabolism with cellular apoptosis in GCs. In bovine GCs, it was observed that excessive non-esterified fatty acids (NEFAs), especially saturated fatty acids (SFAs), could induce apoptosis (Vanholder et al., 2005), and it was also demonstrated in human GCs that SFAs have a positive effect on the apoptotic events (Mu et al., 2001). Significantly, downregulation of *FASN* also induced apoptosis in bovine GCs (Elis et al., 2015). Despite above efforts, the correlation of lipid metabolism with GC functions including steroidogenesis and apoptosis still seems to be far more intricate and remains largely unknown; further investigations are hence required.

In the present study, goose (*Anser cygnoides*) GCs were used as the research model to initially investigate the effects of manipulated *FASN* expression via the construction of the eukaryotic expression vector and RNA interference on lipid metabolism using laser scanning confocal microscopy (LSCM). Then, the effects of *FASN*-mediated lipid metabolism on GC steroidogenesis and apoptosis were determined using the ELISA and flow cytometry (FCM) methods, respectively. Finally, the differences and similarities of these effects were compared between goose ovarian pre-hierarchical GCs (phGCs) and hierarchical GCs (hGCs). These data are expected to provide new insights into the role of *FASN*-mediated lipid metabolism in regulating GC steroidogenesis and apoptosis.

## Materials and Methods

### Experimental Animals

The maternal line of Tianfu meat geese, 35–40 weeks of age and laying in regular sequences of at least two to three eggs, was selected for this study. These geese were kept under the same conditions of light and temperature and were allowed *ad libitum* to feed and water at the Waterfowl Breeding Experimental Farm at Sichuan Agricultural University (Sichuan, China). Individual laying cycles were monitored and recorded, and the healthy geese were euthanized by cervical dislocation 6–8 h ahead of oviposition.

### Preparation of Recombinant Plasmid *pEGFP-N1-FASN* and *FASN*-Targeted siRNA

Specific primers for recombinant plasmid *pEGFP-N1-FASN* construction and siRNA-*FASN* were designed according to the sequence of *FASN* (XM_013197939.1) available in GenBank. Three individual target fragments of *FASN* were amplified under the guidelines of PrimeSTAR Max Premix (TaKaRa, Dalian, China) and then ligated into the *pEGFP-N1* vector previously linearized by *Bgl*II (NEB, Schwalbach, Germany) and *Hin*dIII (NEB, Schwalbach, Germany) with the In-Fusion@ HD Cloning Kit (Clontech, Mountain View, CA, United States) according to the manufacturer’s instructions. Finally, the recombinant plasmid *pEGFP-N1-FASN* was confirmed by restricted enzyme digestion, PCR, and sequencing. Detailed information on the primers of recombinant plasmid construction is summarized in Table 1. siRNA-*FASN*-1 (5′ GCUGGAUGCCAAUAGCUUUTT 3′) and siRNA-*FASN*-2 (5′ GCUCGAUACCUUCCUGAAUTT 3′) were selected from three individual siRNAs due to their most efficiency in reducing *FASN* mRNA expression of phGCs and hGCs separately. In addition, a non-targeting (scrambled) siRNA was used as a control. Moreover, all siRNAs were designed and synthesized by Shanghai GenePharma Company (China).

**TABLE 1:** Specific primers for amplification of *FASN* and construction of *pEGFP-N1-FASN*.

Genes	Accession number	Primers (5′–3′)	Tm (°C)	Size (bp)
*FASN*-1	XM_013197939.1	F:agcgctaccggactcagatctATGGAGGACGTGGTGATTGCA R:TCCTAGCCAGATCACTTTGCCA	60	2197
*FASN*-2	XM_013197939.1	F:GCAAAGTGATCTGGCTAGGAATTC R:GTGTAGAAGTGCTGAAGTGGGGAA	60	2516
*FASN*-3	XM_013197939.1	F:CCACTTCAGCACTTCTACACAACTAA R:cgactgcagaattcgaagcttTTAACCCTCTCTGACACTGACACGT	60	2897

**FASN*-1-forward primer was introduced with the *Bgl*II restriction site (lowercase and underlined part) and 15-bp extensions homologous to the linearized *pEGFP-N1* vector ends. The *FASN*-3-reverse primer was introduced with the *Hin*dIII restriction site (lowercase and underlined part) and 15-bp extensions homologous to the linearized *pEGFP-N1* vector ends. The 5′ termini of DNA fragment *FASN*-2 had 19 bp homologous to the 3′ termini of DNA fragment *FASN*-1, while the 3′ termini of DNA fragment *FASN*-2 had 19 bp homologous to the 5′ termini of DNA fragment *FASN*-3.*

### GCs Culture and Transfection

The granulosa layers separating from pre-hierarchical (6–10 mm) and hierarchical follicles (F4–F1) were washed with PBS (Solarbio, Beijing, China), respectively. GCs were isolated by 0.1% collagenase II digestion, then counted and further cultured into a 12-well culture plate or glass bottom cell culture dish, as previously described (Deng et al., 2018). At 70–80% confluence, GCs were transfected with overexpression plasmid (2 μg/well of a 12-well plate) and siRNA (90 pmol/well of a 12-well plate), respectively, using Lipofectamine 3000 (Invitrogen, Carlsbad, CA, United States) according to the manufacturer’s instructions and were assayed at 48 h post-transfection.

### BODIPY 493/503 Staining of Lipid Droplets (LDs) for Morphological Observation by LSCM and Quantification by FCM

The GCs growing on the glass bottom cell culture dish were fixed with 4% paraformaldehyde at room temperature for 30 min and then incubated in BODIPY (Thermo Fisher, Carlsbad, CA, United States) staining solution (1 μg/ml, diluted in PBS) in the dark at 25°C for 15 min to label the LDs. Subsequently, GCs were washed three times with PBS and then the nuclei were labeled with Hoechst staining solution (2 μg/ml, diluted in PBS) for 15 min. After that, GCs were washed for the final three times with PBS and incubated in 1 ml PBS. Subsequently, the morphological characteristics of LDs were observed with a FV1200 LSCM (Olympus, Japan).

In addition, cell suspension was prepared by digestion with 0.25% trypsin-EDTA (Gibco, Carlsbad, CA, United States), then washed one time with PBS. The cells were further incubated in BODIPY staining solution (100 ng/ml, diluted in PBS) in the dark at 25°C for 15 min to label the LDs, then washed three times with PBS, and resuspended with 200 μl PBS. In the immediate aftermath of filtering, the mean fluorescence intensity (MFI) was measured using the BD Accuri C6 flow cytometer (Becton Dickinson, Franklin Lakes, NJ, United States) and analyzed using FlowJo software (Tree Star Inc., Ashland, OR, United States); 20,000 cells were analyzed per sample.

### Annexin V-FITC/PI Double Staining in the Detection of Apoptosis by FCM

Cell suspension was prepared by digestion with 0.25% trypsin-EDTA, and then staining was performed with the Annexin V/PI cell apoptosis detection kit (Beyotime Biotech, Nantong, China) according to the manufacturer’s instructions. Apoptotic cells were quantified using the BD Accuri C6 Flow cytometer and analyzed using FlowJo software; 20,000 cells were analyzed per sample, and the apoptotic rate was presented as the sum of early and late apoptosis subpopulations.

### ELISA for Determination of P_4_ Production in the Supernatant Culture Medium

The production of P_4_ in the supernatant culture medium was detected by goose P_4_ (PROG) ELISA Kit (Huding Biotechnology, Shanghai, China) according to the manufacturer’s instructions, and finally the P_4_ production of each well was normalized by the total RNA amount of the same well. The data were expressed as a percentage of P_4_ production by control.

### RNA Extraction and qRT-PCR Analysis

The total RNA was extracted from each sample using TRIzol reagent (Invitrogen, Carlsbad, CA, United States) according to the manufacturer’s instructions, then the RNA quality, purity, and concentration were measured by spectrophotometric absorbance measurement. The cDNA was further synthesized from 1 μg of total RNA using a PrimeScript RT^TM^ Reagent Kit (TaKaRa, Dalian, China), in accordance with the manufacturer’s instructions. The qRT-PCR analysis was conducted using 2 × SYBR Premix Ex Taq II (TaKaRa, Dalian, China). The reaction solution was prepared in a total volume of 12.5 μl containing 1 μl cDNA, 6.25 μl of 2 × SYBR Premix Ex Taq, 4.25 μl of ddH_2_O, and 0.5 μl of each gene-specific primer (10 μM). For each sample, the analysis was conducted in triplicate and normalized to β*-actin* by the 2^–Δ^ ^Δ^ ^Ct^ method (Livak and Schmittgen, 2000). The control was set as one. The primers for qRT-PCR are summarized in Table 2.

**TABLE 2:** Primers for qRT-PCR.

Genes	Accession number	Primers (5′–3′)	Tm (°C)	Size (bp)
*FASN*	XM_013197939.1	F:TGGGAGTAACACTGATGGC R:TCCAGGCTTGATACCACA	60	109
*PPAR*α	KJ010765.1	F:ATCTATCCCTGGCTTCTCCA R:AGCATCCCATCCTTGTTCATT	55	117
*CPT-1*	XM_013195075.1	F:GTCTCCAAGGCTCCGACAA R:GAAGACCCGAATGAAAGTA	56	193
*SCD1*	HQ197924.1	F:GCCATCGGTCCTACAAAGC R:AGCCAATGTGGGAGAAGAAA	60	180
*DGAT2*	KF460563.1	F: CGCCATCATCATCGTGGT R:CGTGCCGTAGAGCCAGTTT	60	113
*APOB*	XM_013194833.1	F: CTCAAGCCAACGAAGAAG R: AAGCAAGTCAAGGCAAAA	56	153
*StAR*	XM_013194444.1	F:AGAATCTTGACCTCTTTGACGCTG R:GAGACGGTGGTGGATAACGGA	60	87
*CYP11A1*	KY463321.1	F:AGGGAGAAGTTGGGTGTCTACGA R:CGTAGGGCTTGTTGCGGTAGT	60	89
*3*β*HSD*	KC310447.1	F:GACCTGGGGTTTGGAATTGAG R:TAGGAGAAGGTGAATGGGGTGT	60	170
*SREBP2*	EF579754.1	F: GGACAGATGCCAAGATGC R: GGTCAATGCCCTTCAACA	60	150
β-Actin*	M26111.1	F:CAACGAGCGGTTCAGGTGT R:TGGAGTTGAAGGTGGTCTCG	60	92

F: sense primers; R: antisense primers.*House-keeping gene for data normalization.

### Statistical Analysis

All experimental data were subjected to statistical analysis by Student’s *t*-test. All statistical analyses were performed using SPSS software (SPSS Inc., Chicago, IL, United States). Results were presented as the mean ± SEM of three independent experiments. *P* < 0.05 was considered as significant.

## Results

### Efficiency of *FASN* Overexpression and Interference

**FIGURE 1:**
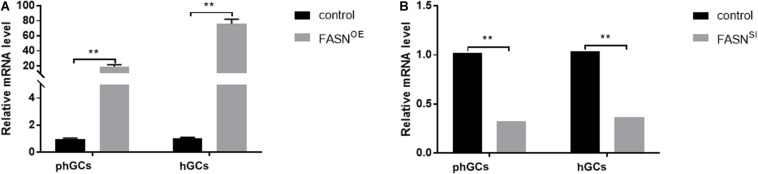
Efficiency of *FASN* overexpression or interference. **(A)** The expression levels of *FASN* of phGCs and hGCs were detected by qRT-PCR 48 h after treatment with *FASN* overexpression, respectively. **(B)** The expression levels of *FASN* of phGCs and hGCs were detected by qRT-PCR 48 h after treatment with *FASN* interference, respectively. OE, overexpression; SI, RNA interference; phGCs, pre-hierarchical GCs; hGCs, hierarchical GCs. The expression of *FASN* was normalized by β*-actin*, and the control was set as one. Results were presented as the mean ± SEM of three independent experiments (**P < 0.01).

Three individual target fragments of *FASN* were obtained by PCR amplification. Then, the recombinant plasmid *pEGFP-N1-FASN* was constructed and confirmed by *Bgl*II and *Hin*dIII double digestion. The expression level of *FASN* after the treatment with *FASN* overexpression and interference was further determined by qRT-PCR. For the overexpression group, the expression levels of *FASN* increased to almost 19- and 76-fold compared to the control (untransfected cells) in phGCs and hGCs, respectively (Figure 1A). In addition, it was worth mentioning that the empty *pEGFP-N1* vector had no influence on the cell viability and FASN expression level of phGCs and hGCs (Supplementary Figure S1). For the interference group, the expression levels of *FASN* reduced to 0.33- and 0.37-fold compared to the control in phGCs and hGCs, respectively (Figure 1B).

### Effects of Overexpressing and Interfering *FASN* on Lipid Accumulation in phGCs and hGCs

**FIGURE 2:**
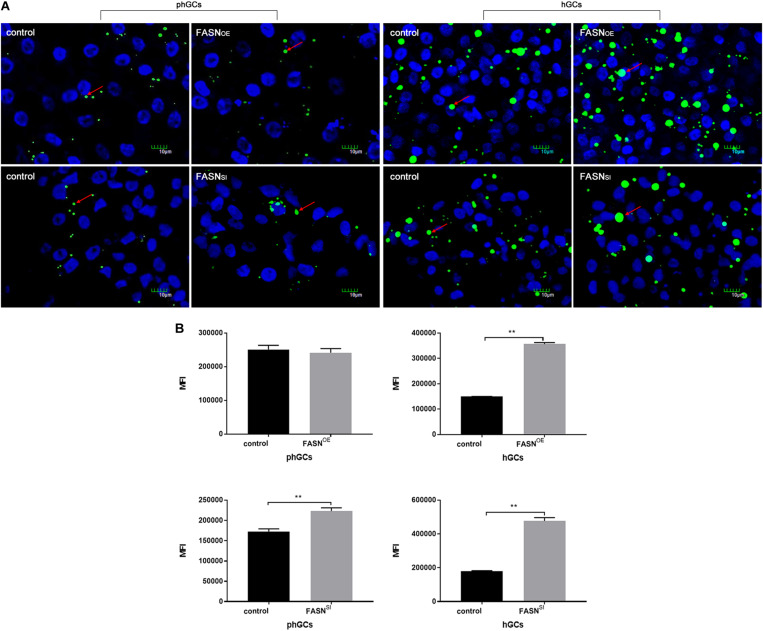
Effects of FASN overexpression or interference on lipid accumulation in cultured phGCs and hGCs. **(A)** Morphological characteristics of phGCs and hGCs were observed by LSCM 48 h after treatment with FASN overexpression and interference, respectively. The red arrows represent LDs. The scale marker represents 10 μm. **(B)** MFI of phGCs and hGCs was determined by FCM 48 h after treatment with FASN overexpression and interference, respectively. 20,000?cells per sample were considered in the gated regions for calculations. OE, overexpression; SI, RNA interference; phGCs, pre-hierarchical GCs; hGCs, hierarchical GCs; MFI, mean fluorescence intensity; FCM, flow cytometry; LSCM, laser scanning confocal microscopy. Results were presented as the mean ± SEM of three independent experiments (**P < 0.01).

The morphological characteristics (Figure 2A) and quantification of LDs (Figure 2B) were measured by LSCM and FCM, respectively. When *FASN* was overexpressed, compared to the control, the content of LDs increased significantly in hGCs (*P* < 0.01) whereas no significant change was observed in phGCs (*P* > 0.05). When *FASN* was interfered, compared to the control, the content of LDs increased significantly in both phGCs and hGCs (*P* < 0.01).

### Effects of Overexpressing and Interfering *FASN* on Apoptosis of phGCs and hGCs

**FIGURE 3:**
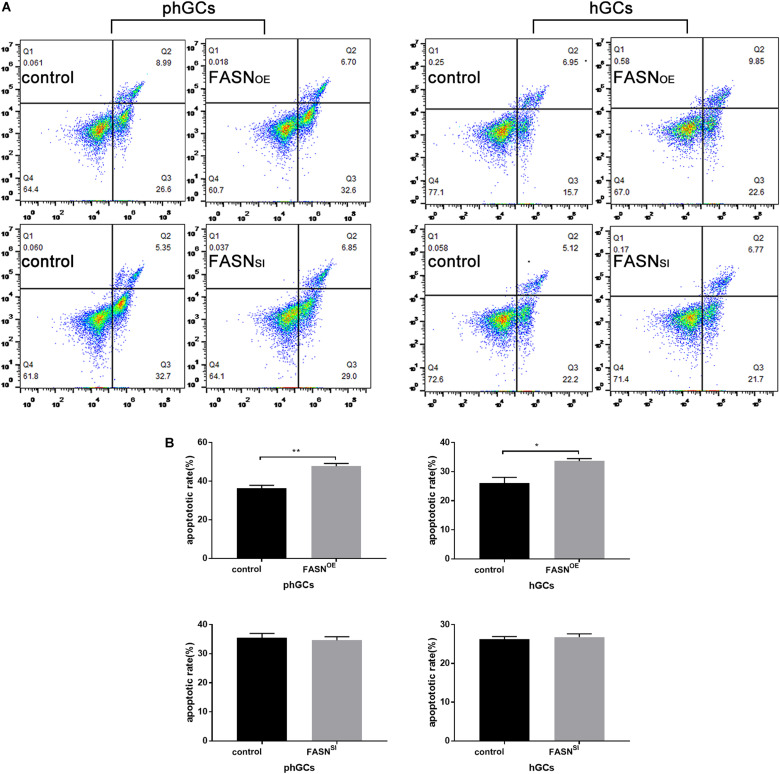
Effects of *FASN* overexpression or interference on apoptosis of cultured phGCs and hGCs. **(A)** Apoptosis of phGCs and hGCs was determined by FCM 48 h after treatment with *FASN* overexpression and interference, respectively. The upper-left (Q1), upper-right (Q2), lower-right (Q3), and lower-left (Q4) quadrants in each panel represent the populations of cell debris, late apoptotic and necrotic, early apoptotic, and normal cells, respectively. **(B)** The overall apoptotic rate was the sum of early and late apoptosis subpopulations. 20,000?cells per sample were considered in the gated regions for calculations. OE, overexpression; SI, RNA interference; phGCs, pre-hierarchical GCs; hGCs, hierarchical GCs; FCM, flow cytometry. Results were presented as the mean ± SEM of three independent experiments (*P < 0.05, **P < 0.01).

The apoptosis was determined by FCM, and the results are displayed in Figures 3A,B. Overexpressing *FASN* significantly increased the apoptotic rate of both phGCs (*P* < 0.01) and hGCs (*P* < 0.05); however, interfering *FASN* showed no significant effect on apoptosis in either phGCs or hGCs (*P* > 0.05).

### Effects of Overexpressing and Interfering *FASN* on P_4_ Production of phGCs and hGCs

**FIGURE 4:**
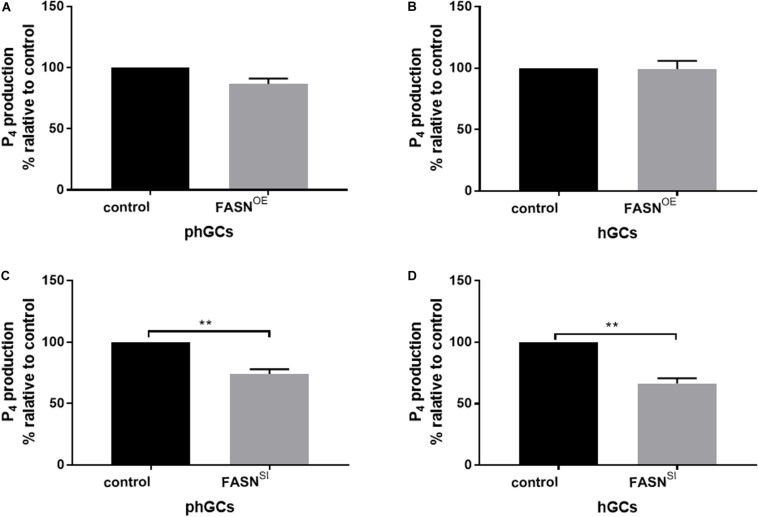
Effects of *FASN* overexpression or interference on P_4_ production of cultured phGCs and hGCs. P_4_ production of phGCs **(A)** and hGCs **(B)** was determined by the ELISA method 48 h after treatment with *FASN* overexpression, respectively. P_4_ production of phGCs **(C)** and hGCs **(D)** was determined by the ELISA method 48 h after treatment with *FASN* interference as determined by the ELISA method, respectively. OE, overexpression; SI, RNA interference; phGCs, pre-hierarchical GCs; hGCs, hierarchical GCs; P_4_, progesterone. P_4_ production was normalized by the total RNA amount of the well and expressed as a percentage of P_4_ production by control. Results were presented as the mean ± SEM of three independent experiments (**P < 0.01).

The production of P_4_ in the supernatant culture medium was determined by the ELISA method, and the results are displayed in Figure 4. Overexpressing *FASN* had no significant effect on P_4_ production in both phGCs (*P* > 0.05) (Figure 4A) and hGCs (*P* > 0.05) (Figure 4B), while interfering *FASN* significantly reduced the P_4_ production in both phGCs (*P* < 0.01) (Figure 4C) and hGCs (*P* < 0.05) (Figure 4D).

### Effects of Overexpressing and Interfering *FASN* on the Expression Levels of Genes Involved in Lipid Metabolism and Steroidogenesis

**FIGURE 5:**
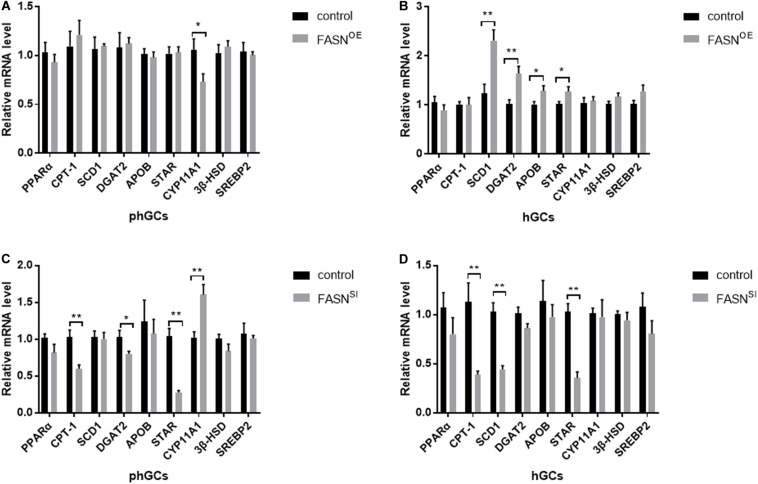
Effects of *FASN* overexpression or interference on the expression levels of lipid metabolism- and steroidogenesis-related genes of cultured phGCs and hGCs. The expression levels of lipid metabolism- and steroidogenesis-related genes of phGCs **(A)** and hGCs **(B)** were detected by qRT-PCT 48 h after treatment with *FASN* overexpression, respectively. The expression levels of lipid metabolism- and steroidogenesis-related genes of phGCs **(C)** and hGCs **(D)** were detected by qRT-PCT 48 h after treatment with *FASN* interference, respectively. OE, overexpression; SI, RNA interference; phGCs, pre-hierarchical GCs; hGCs, hierarchical GCs. The expression of genes was normalized by β*-actin*, and the control was set as one. Results were presented as the mean ± SEM of three independent experiments (*P < 0.05, **P < 0.01).

The expression levels of lipid metabolism- and steroidogenesis-related genes were assessed by qRT-PCR, and the results are displayed in Figure 5. When *FASN* was overexpressed, in phGCs (Figure 5A), the expression levels of perixisome proliferation-activated receptor alpha (*PPAR*α), carnitine palmitoyltransferase 1 (*CPT-1*), stearoyl-CoA desaturase 1 (*SCD1*), diacylglycerol acyltransferase 2 (*DGAT2*), apolipoprotein B (*APOB*), steroidogenic acute regulatory (*StAR*) protein, 3β-hydroxysteroid dehydrogenase (*3*β*HSD*), and sterol regulatory element binding protein-2 (*SREBP2*) were not significantly affected (*P* > 0.05), whereas the expression level of recombinant cytochrome P450 11A1 (*CYP11A1*) was reduced significantly (*P* < 0.05); in hGCs (Figure 5B), the expression levels of *SCD1* (*P* < 0.01), *DGAT2* (*P* < 0.01), *APOB* (*P* < 0.05), and *StAR* (*P* < 0.05) were increased significantly, while the expression levels of *PPAR*α, *CPT-1*, *CYP11A1*, *3*β*HSD*, and *SREBP2* were not significantly affected (*P* > 0.05). When *FASN* was interfered, in phGCs (Figure 5C), the expression levels of *CPT-1*(*P* < 0.01), *DGAT2* (*P* < 0.05), and *StAR* (*P* < 0.01) were decreased significantly, whereas the expression levels of *CYP11A1* were increased significantly (*P* < 0.01), and no significant effects were seen in the expression levels of *PPAR*α, *SCD1*, *APOB*, *3*β*HSD*, and *SREBP2* (*P* > 0.05); in hGCs (Figure 5D), the expression levels of *CPT-1*, *SCD1*, and *StAR* were reduced significantly (*P* < 0.01), while the expression levels of *PPAR*α, *DGAT2*, *APOB*, *CYP11A1*, and *3*β*HSD* and *SREBP2* were not significantly affected (*P* > 0.05).

## Discussion

To our knowledge, this study represents the first to determine the effects of *FASN* on lipid metabolism of phGCs and hGCs and to explore the influence of *FASN*-mediated lipid metabolism on GC steroidogenesis and apoptosis in geese. Previous studies have indicated that upregulation of *FASN* induced increased lipid accumulation in bovine (Chu et al., 2018b) and murine (Chu et al., 2018a) mammary epithelial cells. Consistent with this, our data also showed that overexpressing *FASN* increased the amount of LDs in goose hGCs although it appeared to have no effect on those in phGCs. Furthermore, overexpressing *FASN* increased the mRNA levels of *SCD1* and *DGAT2* (two key regulatory enzymes in DNL) in hGCs instead of phGCs. It has been reported that once palmitic acid is obtained, it can be de-saturated by *SCD1* (Strable and Ntambi, 2010). These unsaturated fatty acids (USFAs) can be further esterified into triglyceride (TG) through the catalysis of *DGAT2* and then be stored into LDs (Ameer et al., 2014). Thus, it would be understandable that overexpression of *FASN* failed to promote lipid accumulation in phGCs because it did not induce similar changes in expression of DNL-related genes in phGCs to those in hGCs. From Figure 5B, overexpressing *FASN* also increased the mRNA levels of *APOB* (a gene participated in the regulation of lipoprotein assembly and secret) in hGCs, from which we inferred that the dramatically increased deposition might promote the transport of lipids from intracellular to the extracellular, which would affect the content of yolk lipids.

In addition, a previous study showed that downregulation of *CPT-1* (a key regulatory enzyme in the β-oxidation of long-chain fatty acids) induced an increase in intracellular lipid content of porcine adipocyte (Zhang et al., 2014). Our results showed that in both phGCs and hGCs, the significantly increased amount of LDs was accompanied by the decrease in *CPT-1* when *FASN* was interfered and which was similar to the previous report. Free fatty acids (FFAs) are the endogenous ligand of *PPAR*α, and the receptor ligand complexes can further activate *CPT-1* expression (Huang et al., 2012). After prolonged fasting, FASKOL (*FASN* knockout in liver) mice developed fatty liver, similarly to fasted *PPAR*α-deficiency mice (Chakravarthy et al., 2005). However, the interference of *FASN* did not affect the expression of *PPAR*α in this study. It indicated that the reduction in *PPAR*α ligand instead of *PPAR*α caused by *FASN* downregulation induced an inhibition of the *PPAR*α-*CPT-1* pathway, which should be responsible for the marked increase in lipid accumulation after downregulating *FASN* in our study. Moreover, the expression of *DGAT2* in the two categorized GCs showed a decreasing trend although the decrease was not significant in hGCs. Thus, we speculated that the increasing content of LDs was irrelevant to the genes related to DNL when *FASN* was interfered. Taken together, the mechanism of regulating lipid metabolism by *FASN* in phGCs was inconsistent with that in hGCs. Specifically, *FASN* could participate in regulating the lipid content in phGCs mostly by activating the *PPAR*α-*CPT-1* pathway but could regulate it in hGCs by activating both the DNL (*FASN*-*SCD1/DGAT2/APOB*) pathway and the lipid oxidation (*PPAR*α-*CPT-1*) pathway.

Saturated fatty acids could induce apoptosis not only in GCs but also in other cell types (Jorritsma et al., 2004; Vanholder et al., 2005, 2006). In this study, the apoptotic rate of both phGCs and hGCs significantly increased by overexpression of *FASN* rather than interference. Previous studies reported that the conversion of SFAs into USFAs by *SCD1* in cumulus cells (a type of GC) protected cells against lipid-induced damage, such as apoptosis, through promoting the distribution of fatty acids toward LDs (Listenberger et al., 2003; Aardema et al., 2017). *SCD1* provides a more accessible pool of USFAs for *DGAT2* for TG synthesis through substrate channeling (Man et al., 2006). As discussed earlier, overexpressing *FASN* had no significant effects on the amount of LDs in phGCs, as well as the expression of *SCD1* and *DGAT2*. This led to a conclusion that phGCs were negatively affected by the dramatically increased but not effectively de-saturated fatty acids caused by *FASN* overexpression, and which finally induced the increase of apoptosis. However, overexpressing *FASN* increased *SCD1* and *DGAT2* gene expression of hGCs to about 2-fold and 1.6-fold, respectively, and the interaction of these two enzymes could protect hGCs against lipid-induced damage. However, the apoptosis of hCGs still increased, though in a less intense form compared to that in phGCs. Considering that the expression of *FASN* increased 76-fold after overexpressing, the slightly increasing *SCD1* and *DGAT2* appeared to be inadequate to totally convert the massive SFAs to USFAs and then to TG. It indicated that hGCs were capable of preventing themselves from apoptosis induced by lipid metabolism dysfunction through activating *SCD1* and *DGAT2* for finally channeling SFAs into TG pools and storing into LDs. *FASN* is preferentially overexpressed in cancer cells and has been strongly linked to cancer cell proliferation and migration (Jiang et al., 2012). Plenty of previous studies on cancer cells showed that inhibiting *FASN* can increase apoptosis (Gonzalezguerrico et al., 2008; Richa et al., 2015; Sun et al., 2018; You et al., 2019). However, the demands of phGCs and hGCs for *FASN* are obviously lower than those of cancer cells, so the sensitivity of GCs to *FASN* inhibition may also be lower than that of cancer cells. Taken together, our results suggested that although the apoptosis induced by *FASN*-mediated lipid metabolism in the two categorized GCs was similar, expressions of *SCD1* and *DGAT2* were positively correlated with those of *FASN* in hGCs but not in phGCs. This difference might indicate that, during early stages of follicle development, GCs could not protect themselves from apoptosis induced by lipid metabolism dysfunction through activating the expression of *SCD1* and *DGAT2*. Moreover, such capacity would be given to GCs with the development of follicles.

Cholesterol in the cytoplasm can be delivered to the mitochondrial inner membrane by *StAR*, then be converted to pregnenolone by *CYP11A1*, and these pregnenolones are later converted to P_4_ by *3*β*HSD* in mitochondria (Marion and Li, 2008). In this study, P_4_ production and the expression of *StAR* in both phGCs and hGCs were decreased by interference of *FASN* rather than overexpression. It indicated that the inhibition of *FASN* reduced P_4_ production by preventing cholesterol from entering the mitochondrial inner membrane. *In vivo*, reduction in P_4_ has been reported to cause ovulation disorder in avian (Etches, 1979) and fail to protect luteal cells from apoptosis induced by *PRL* (prolactin) in mammals (Okuda et al., 2004). Unlike the interference, the overexpression of *FASN* had no effect on P_4_ production for those two categorized GCs. As shown in Figure 5A, in phGCs, the unchanged expression levels of *StAR*, *CYP11A1*, and *3*β*HSD* might be the reason for the failure of P_4_ production to respond to the increasing fatty acid *de novo* synthesis. In hGCs, although the cholesterol entering the mitochondrial inner membrane probably increased with the increase in *StAR* expression, P_4_ production is still limited by the limited effectiveness of catalysis by *CYP11A1* and *3*β*HSD*, as shown in Figure 5B. Despite that the responses of the steroidogenesis-related gene expression levels in phGCs were not that consistent with those in hGCs for the stimulation of *FASN* overexpression, the result was no effect on P_4_ production in the two categorized GCs. Besides the stimulation of gonadotropins (Rivas et al., 2015), steroidogenesis is also stimulated by other physiological active substances existing in the follicles (Groothuis et al., 2005). According to our results, the increasing fatty acids induced by overexpression of *FASN* were not such substances that could effectively stimulate the steroidogenesis in goose GCs from the follicles at different stages of development. Additionally, we also measured the mRNA levels of *SREBP2*, which is a master regulator of intracellular cholesterol homeostasis. It has been reported that, when cells are deprived of cholesterol, *SREPB2* would be activated then lead to enhanced cholesterol uptake and biosynthesis (Miserez et al., 2002). However, in this study, *SREBP2* was not affected by *FASN* overexpression or interference either in phGCs or hGCs. Thus, it could be inferred that the decrease in P_4_ production in both phGCs and hGCs induced by *FASN* interference is probably not due to the decrease in intracellular cholesterol. However, it has been proved that, in bovine GCs, the reduction in P_4_ biosynthesis by inhibiting *FASN* was likely related to the potential inhibition of cholesterol biosynthesis (Elis et al., 2015). However, our results indicated that, in goose GCs, *FASN* participated in the regulation of steroidogenesis through *StAR* which can modulate the amount of cholesterol that enters the mitochondrial inner membrane. This demonstrated that the ways for *FASN*-mediated lipid metabolism to involve in regulating steroidogenesis of GCs could be various.

## Conclusion

In summary, this study not only examined the effects of manipulated *FASN* expression on lipid metabolism of goose phGCs and hGCs but also explored the effects of *FASN*-mediated lipid metabolism on cellular apoptosis and steroidogenesis. The most interesting finding was that lipid metabolic homeostasis appeared to be one of the prerequisites for GCs to maintain its normal function. Specifically, *FASN*-mediated lipid metabolism can regulate GC apoptosis, which may be related to lipotoxicity; besides, our study provides new mechanistic insights into the regulation of P_4_ production by *FASN*-mediated lipid metabolism, in which the regulation of *StAR* by *FASN* is critical. These findings are expected to provide a theoretical support for further elucidating the mechanism of lipid metabolism in regulating the function of avian GCs throughout follicular development.

## Data Availability Statement

The raw data supporting the conclusions of this article will be made available by the authors, without undue reservation.

## Ethics Statement

All procedures in this study were approved by the Faculty Animal Care and Use Committee of Sichuan Agricultural University (Sichuan, China).

## Author Contributions

XC contributed to methodology, software, validation, formal analysis, investigation, resources, writing—original draft preparation, writing—review and editing, and visualization. KH contributed to software and investigation. SH contributed to conceptualization, data curation, writing—review and editing, funding acquisition, and project administration. GL, XG, SG, YD, JH, LL, BH, HH, HL, and LX contributed to investigation. JW contributed to supervision, project administration, and funding acquisition.

## Conflict of Interest

The authors declare that the research was conducted in the absence of any commercial or financial relationships that could be construed as a potential conflict of interest.

## Supplementary Material

FIGURE S1 Effect of *pEGFP-N1* vector on cell viability and FASN expression level of phGCs and hGCs.
