# Supplementary figures and images for: FASN-Mediated Lipid Metabolism Regulates Goose Granulosa Cells Apoptosis and Steroidogenesis
Source: Front Physiol. 2020 Jun 26;11:600. doi: 10.3389/fphys.2020.00600 (PMC7333536; doi:10.3389/fphys.2020.00600)

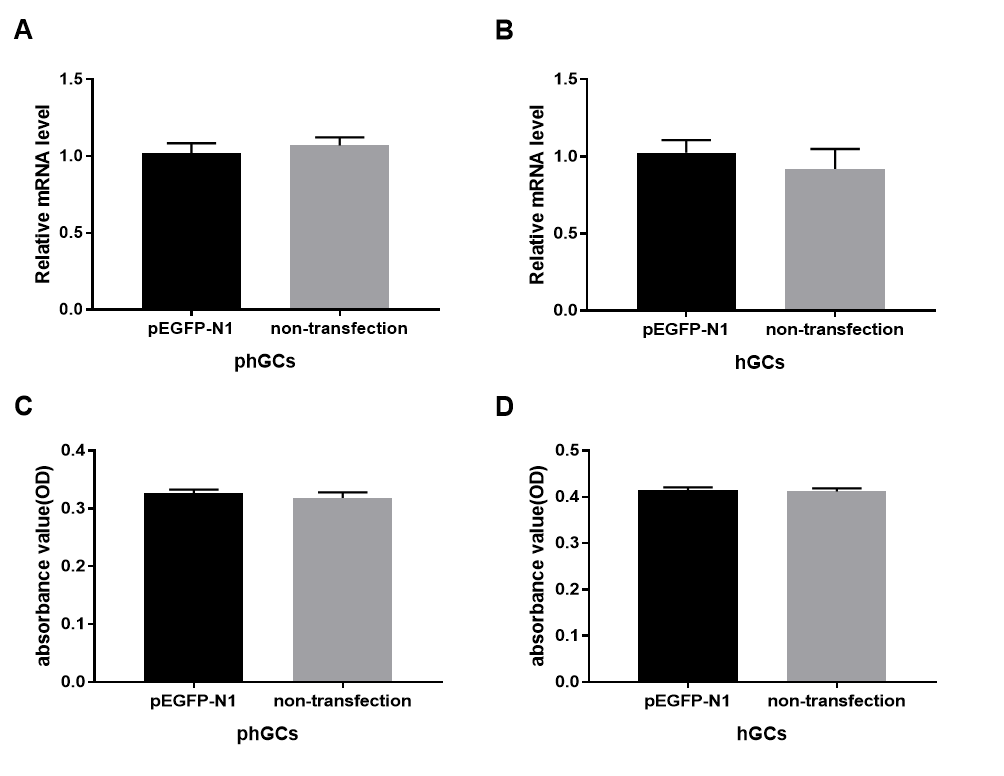

Supplement: FIGURE S1 — Effect of pEGFP-N1 vector on cell viability and FASN expression level of phGCs and hGCs. [file Image_1.TIF]
